# Capacitive Impedance Measurement: Dual-frequency Approach

**DOI:** 10.3390/s19112539

**Published:** 2019-06-04

**Authors:** Alan Kardek Rêgo Segundo, Érica Silva Pinto, Gabriel Almeida Santos, Paulo Marcos de Barros Monteiro

**Affiliations:** 1 Escola de Minas, Universidade Federal de Ouro Preto (UFOP), Morro do Cruzeiro, 35400-000 Ouro Preto, MG, Brazil; erica.lins15@gmail.com (É.S.P.); gabrielalmeida.el@gmail.com (G.A.S.); pmemop@gmail.com (P.M.d.B.M.); 2 Instituto Tecnológico Vale (ITV), Avenida Juscelino Kubitschek, 31, Bauxita, 35400-000 Ouro Preto, MG, Brazil

**Keywords:** dielectric constant, electrical conductivity, instrumentation, microcontroller, embedded system

## Abstract

The most widely used technique for measuring capacitive impedances (or complex electrical permittivity) is to apply a frequency signal to the sensor and measure the amplitude and phase of the output signal. The technique, although efficient, involves high-speed circuits for phase measurement, especially when the medium under test has high conductivity. This paper presents a sensor to measure complex electrical permittivity based on an alternative approach to amplitude and phase measurement: The application of two distinct frequencies using a current-to-voltage converter circuit based in a transimpedance amplifier, and an 8-bit microcontroller. Since there is no need for phase measurement and the applied frequency is lower compared to the standard method, the circuit presents less complexity and cost than the traditional technique. The main advance presented in this work is the use of mathematical modeling of the frequency response of the circuit to make it possible for measuring the dielectric constant using a lower frequency than the higher cut-off frequency of the system, even when the medium under test has high conductivity (tested up to 1220 μS/cm). The proposed system caused a maximum error of 0.6% for the measurement of electrical conductivity and 2% for the relative dielectric constant, considering measurement ranges from 0 to 1220 μS/cm and from 1 to 80, respectively.

## 1. Introduction

Material impedance measurements range from medical applications such as disease diagnosis [1] to agricultural applications, such as estimating soil electrical parameters to automate irrigation systems [2,3,4,5,6,7,8], optimize water resources [9,10,11], biomass flow sensing [12], or detect food quality properties. In addition, there are several other applications in the field of multiphase flow measurement [13,14,15,16], hydrology and process control (e.g., concrete hardening, compost maturation, moisture in bins, etc.).

The main difficulty encountered when measuring the complex electrical permittivity of materials is to separately evaluate the effects of the conductivity (σ) and the relative dielectric constant (ε) of the medium under test, especially when the conductivity is high.

In some applications, the separation of σ and ε effects is not a problem, as the material under test is not too conductive (up to 10 µS) [13,14,15,16,17]. In other ones, σ does not play an important role, as the measurement technique used in Reference [12]. In the last decade, many works show studies about the development of circuits for measuring lossy capacitive sensors [18,19,20], but for applications which don’t have frequency dependence and/or the conductance is not too high (up to 145 µS), such as for measuring gas concentration, humidity, flow, and pressure. However, for applications with frequency dependence, like soils, the choice of frequencies may lead to uncertainties due to many relaxation phenomena. Sen Wang et al. [21] report that the frequency must be higher than 100 kHz, otherwise, the changes are great in soil resistivity and dielectric constant when the frequency is low; and the effect of the moisture content of the soil on the frequency-dependent properties of resistivity and dielectric constant is great.

Chavanne et al. [9] summarized that capacitive sensors currently available for measuring electrical properties of soils are based on the frequency of an electronic LC oscillator, like EnviroSCAN and TriSCAN probes [22]; the root mean square of partial-charging cycles of a capacitor, like Decagon probes, described in Reference [23]; or the propagation and reflection of a sinusoidal wave in a coaxial line ended by the electrodes, such as Hydra Probe and Theta Probe [24,25]. These techniques make their physical modeling difficult, thus they use some empirical conversion to obtain ε, and/or σ cannot be measured with the same technique as for ε, or with poor accuracy.

Other research shows the development of systems based on the standard method [1,10,11,26,27], which requires a more complex circuit, faster hardware, and more signal processing.

In [2], we presented a low-cost system to measure the complex electrical permittivity of the soil using lower frequencies compared to the standard method (100 kHz and 5 MHz), aiming to automate irrigation systems. However, Chavanne et al. [9] questioned the system’s ability to separately measure σ and ε of the medium under test if the conductivity is greater than 70 μS/cm, since the frequencies used were very low (100 kHz and 5 MHz). In Reference [28], we proposed increasing the frequency of the system using the same electronic design presented in Reference [2], but with a higher speed microcontroller.

The objective of this work is to use the mathematical modeling of the system in the frequency domain to minimize the effect that σ exerts under ε, and vice versa, even with lower frequencies in relation to the standard method, in order to perform non-biased capacitive impedance measurements.

In the next section, we present the theory involving capacitive impedance measurement. In Section 3, we show the materials and methods adopted in this work. Section 4 presents achieved results and discussions. In Section 5, we point out the main conclusions of this work.

## 2. Theory

The materials can be characterized by its electrical properties. The complex relative permittivity (**ε**) is related to the absolute complex permittivity (**ε_a_**) according to Equation (1), and it is used in this work to characterize the capacitive impedance:

(1) $$\varepsilon_{a}=\varepsilon_{0}\;\varepsilon$$

where ε_0_ is the permittivity of free space (=8.854 pF·m^−1^).

Working with complex relative permittivity needs attention at GHz-range, where a phenomenon called dielectric relaxation occurs, i.e., the real part of **ε** decreases with increasing frequency. Since this work uses measurements limited to 8 MHz, relaxation mechanisms can be neglected, and the complex relative permittivity can be represented by:

(2) $$\varepsilon =\varepsilon -j\frac{\sigma }{{\omega \varepsilon }_{0}}$$

where ε is the relative dielectric constant (also known as dielectric, dimensionless), $j=\sqrt{-1}$, σ the electric conductivity (S·m^−1^) and ω the angular velocity (rad·s^−1^). The imaginary part is the dielectric losses factor.

The impedance (**Z**) of a material corresponds to the ratio between the voltage (**V**) and the current (**I**) phasors, according to Ohm’s law in complex notation, that is:

(3) Z=R+jX

where *R* is the resistance (Ω) and *X* the reactance (Ω). The real parts of (1) and (2) are related to the losses by Joule effect. The imaginary part is the ability to exchange energy.

The impedance measurements of solid, liquid or gaseous substances are carried out by means of a probe. However, to simplify the electrical circuit analysis in the case of capacitive impedance, it is more convenient to use the admittance (**Y**)—inverse of impedance:

(4) Y=G+jB

where *G* is the conductance (S) and *B* the susceptance (S).

By replacing (1) and (2) in (4), we have the admittance of a substance related to the electrical property **ε**:

(5) $$Y=j\omega k_{g}\varepsilon_{0}\varepsilon$$

where *k_g_* is the geometric constant of the probe, $G=k_{g}\sigma$ and $B=k_{g}{\omega \varepsilon }_{0}\varepsilon$.

In this work we used a sensor of cylindrical rods and parallel to each other, which presents geometric constant:

(6) $$k_{g}=\frac{\pi l}{\ln\left(\frac{d}{2a}+\sqrt{\frac{d^{2}}{4a^{2}}-1}\right)}$$

where *l* is the length (m), *d* is the distance between the axes (m) and *a* is the radius of the rods (m).

In Reference [2], we discussed that, as the impedance is a complex variable, it is necessary to determine at least two parameters to define it: Module and phase, or real and imaginary part of voltage or current. The standard method to analyze the impedance of materials consists on applying a pure sinusoidal voltage at a single frequency to the sensor electrodes and measure the phase shift and amplitude, or the real and imaginary parts, of the resulting current using either analog circuit or analog-to-digital conversion and signal processing algorithm to analyze the response [13,15].

In Reference [28], we reviewed the methods for measuring impedance at low frequency (up to the MHz band) and classified them into three categories: Current-to-voltage (or I-V), bridge, or resonant methods. The I-V method is very simple, however with low accuracy. The classical bridge method has high accuracy, but due to the need for balancing, it is not suitable for fast and continuous measurements. More recently, Chavanne et al. [27] were able to circumvent these limitations. The last one has good accuracy, but similarly to the bridge method, as it needs tuning, is not suitable for fast measurements.

**Figure 1:**
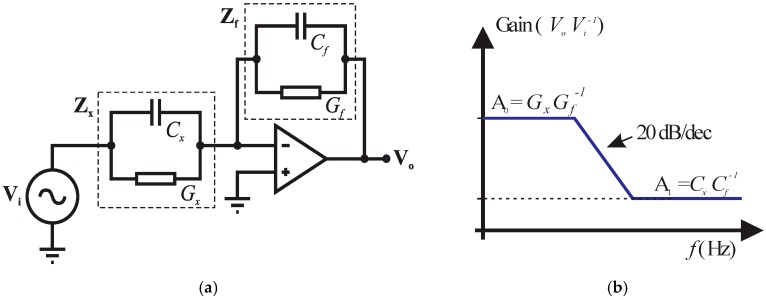
(**a**) I-V converter circuit and (**b**) frequency domain response.

Therefore, we proposed a variation of the I-V impedance measuring method [2] in order to design a simple circuit with low cost, high accuracy and fast response. The addition of an operational amplifier with high input and low output impedance provides a high signal to noise ratio and stray capacitance immunity, capable of measuring small impedances between the electrodes even in the presence of large stray capacitances to ground [15,29]. This setting is also known as transimpedance amplifier or current-to-voltage converter, as shown in Figure 1.

It is noteworthy that capacitive sensors can be classified in two types: Floating and grounded [18]. In the first type, the two terminals of the sensor are available to the measure circuit, instead in the second one of the terminals is always at ground potential. In this work, we use a floating type measuring circuit, as shown in Figure 1.

The basic circuit diagram is represented in Figure 1a, where **V_i_** is the input voltage, **Z_x_** is the unknown impedance, **Z_f_** is the impedance of feedback circuit and **V_o_** is the output voltage. The operational amplifier non-inverting port is connected to ground. Thus, the currents passing through **Z_f_** and **Z_x_** are balanced through the operational amplifier action (i.e., the sum of the currents in the virtual ground is considered to be zero) and the current through the unknown impedance is proportional to the operational amplifier output voltage.

The connection of the sensor (represented by **Z_x_**) in the circuit through cables can create some stray capacitances *C*_*s*1_ and *C*_*s*2_. These capacitances do not affect the measurements since *C*_*s*1_ is drained directly by the voltage source and *C*_*s*2_ is virtually grounded by the operational amplifier. This is a major advantage in this circuit [15]. The gain of the circuit shown in Figure 1b is given by:

(7) $$\frac{V_{o}}{V_{i}}=-\frac{Z_{f}}{Z_{x}}=-\frac{Y_{x}}{Y_{f}}=-\left(\frac{G_{x}+j\;\omega C_{x}}{G_{f}+j\;\omega C_{f}}\right)=-k_{g}\left(\frac{\sigma +j\;{\omega \varepsilon }_{0}\varepsilon }{G_{f}+j\;\omega C_{f}}\right)$$

where *G_x_* and *G_f_* are the conductances (S), *C_x_* and *C_f_* are the capacitances (F) of the impedances **Z_x_** and **Z_f_**, respectively.

In (7), *G_x_* and *C_x_* are directly related to the determination of σ and ε. This determination involves the measurement at least of two parameters, which may be: (i) magnitude and phase in a single frequency; (ii) real and imaginary parts of **V_o_** at a single frequency; or (iii) two amplitude gains at different frequencies. These three possibilities are mathematically equal, but differ with respect to the complexity of the circuit [15,30]. This work proposes an electronic circuit for instrumentation that uses the third option to estimate σ and ε of the material located between the probe electrodes, i.e., two gains are measured by the sensor, *A*_0_ = *V_o_*/*V_i_* and *A*_1_ = *V_o_*/*V_i_*, using two different frequencies equals to 500 kHz and 8 MHz, respectively.

**Figure 2:**
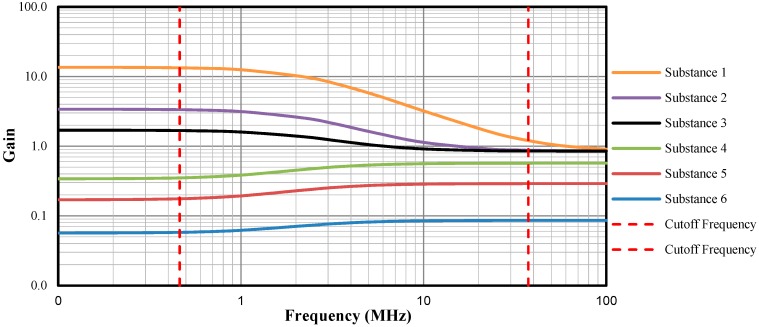
System frequency response for different σ and ε (*R_f_* = 680 Ω, *C_f_* = 100 pF, *k_g_* = 0.122 m, σ_1_= 1645.4 µS/cm, σ_2_ = 411.5 µS/cm, σ_3_ = 205.7 µS/cm, σ_4_ = 41.1 µS/cm, σ_5_ = 20.6 µS/cm, σ_6_ = 6.9 µS/cm, ε_1_ = ε_2_ = ε_3_ = 80, ε_4_ = 53, ε_5_ = 27, and ε_6_ = 8).

Figure 2 shows a frequency response simulation of the system for 6 different substances, as an example. Each curve has two cut-off frequencies ($f={\left(2\pi RC\right)}^{-1}$), delimited between dashed red lines drawn vertically: the first one depends on *G_f_* and *C_f_* of the fixed reference impedance **Z_f_**; and the second depends on *G_x_* and *C_x_* of the impedance **Z_x_**, measured by the sensor. Depending on the electrical properties of the material, the second cut-off frequency may exceed the value of the sensor’s power signal frequency and, consequently, the estimate of *C_x_* becomes biased, since the frequency of the second plateau cannot be generated by the microcontroller. In Figure 2, it can be observed, for example, that substances 3, 4, 5, and 6 strike the second plateau at much lower frequencies than substances 1 and 2, that is, the higher the conductivity of a substance the greater the frequency of the second plateau.

In Reference [2], the frequency approximation *f* → 0 was used to estimate the conductance *G_x_* and *f* → ∞ to estimate the capacitance *C_x_* since the gains *A*_0_ and *A*_1_ at each plateau of the frequency response curve (Figure 1b) are equal to *G_x_G_f_*^−1^ and *C_x_C_f_*^−1^, respectively. However, this approach has the limitation of not independently measuring the effects of conductance and capacitance when the conductivity of the material under test is high, because the frequency of the sensor’s signal source may not be high enough to reach the second plateau of Figure 1b.

Therefore, in this work, to circumvent this limitation, instead of using the approximations *f* → 0 and *f* → ∞, we propose a method called real-dual-frequency, which take into account the real frequencies of the two signals applied in **V_i_**, thus, from (7):

(8) $$A_{0}=\sqrt{\frac{G_{x}^{2}+\;\omega_{0}^{2}C_{x}^{2}}{G_{f}^{2}+\;\omega_{0}^{2}C_{x}^{2}}}$$

(9) $$A_{1}=\sqrt{\frac{G_{x}^{2}+\;\omega_{1}^{2}C_{x}^{2}}{G_{f}^{2}+\;\omega_{1}^{2}C_{x}^{2}}}$$

Then, since *A*_0_ and *A*_1_ are measured by the sensor and *ω*_0_, *ω*_1_, *G_f_* and *C_f_* are known, by manipulating (8) and (9), we obtain the expressions to estimate σ and ε separately, under the hypotheses that effect of the conductivity on the dielectric constant measurement, and vice versa, will be minimized, even without reaching the frequency of the second plateau:

(10) $$\sigma =\frac{1}{k_{g}}\sqrt{\frac{A_{1}^{2}\omega_{0}^{2}(G_{f}^{2}+\omega_{1}^{2}C_{f}^{2})-A_{0}^{2}\omega_{1}^{2}(G_{f}^{2}+\omega_{0}^{2}C_{f}^{2})}{\left(\omega_{0}^{2}-\omega_{1}^{2}\right)}}$$

(11) $$\varepsilon =\frac{1}{\varepsilon_{0}k_{g}}\sqrt{\frac{A_{0}^{2}(G_{f}^{2}+\omega_{0}^{2}C_{f}^{2})-A_{1}^{2}(G_{f}^{2}+\omega_{1}^{2}C_{f}^{2})}{\left(\omega_{0}^{2}-\omega_{1}^{2}\right)}}$$

## 3. Methodology

### 3.1. Sensor and Measurement Circuit

**Figure 3:**
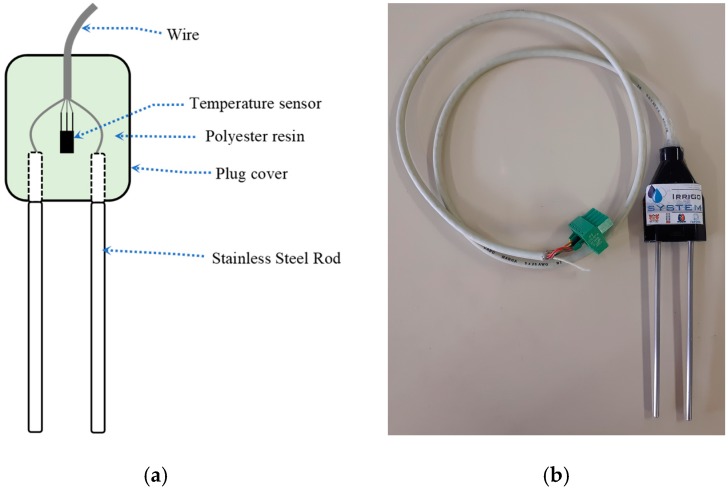
(**a**) Scheme of the probe for measuring σ and ε; (**b**) Probe used in the experiments.

In this work, the probe and the measurement circuit were developed based on the same methodology presented in [2]. We constructed the probes using stainless steel rods (length = 155 mm, being 97 mm exposed, diameter = 3 mm, spaced of 16.8 mm to one another; thus, *k_g_* = 0.13 m), liquid polyester resin, a semiconductor temperature sensor (LM35), five-way cable (length = 1 m) and covers for electrical outlet plugs, according to the scheme shown in Figure 3a. The covers for electrical outlet plugs served to fix the rods and the temperature sensor using polyester resin. Figure 3b illustrates the probes implementation.

**Figure 4:**
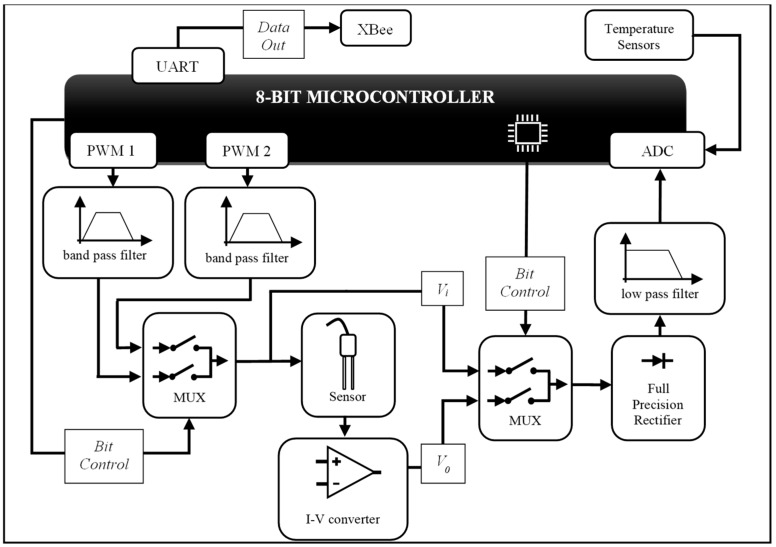
Schematic diagram of the sensor measurement circuit.

The operational diagram of the measuring circuit is shown in Figure 4. For this implementation, the I-V converter uses a 680 Ω resistor and 100 pF capacitor for **Z_f_**. The microcontroller generates two PWM signals of 500 kHz and 8 MHz frequencies with a 50% duty cycle to provide the input signals (*V_i_*) to the sensor. Two filters are used to transform the square wave of PWM signals into sinusoidal signals. An analog multiplexer is used to select between 500 kHz or 8 MHz input signals.

The sensor is modeled as a capacitor in parallel with a resistor, which forms the unknown impedance **Z_x_** of the current-to-voltage conversion circuit based on a transimpedance amplifier (see details in Figure 1). A second analog multiplexer is used to select which signal will be measured by the ADC, *V_i_* or *V_o_*, after passing through the full precision rectifier and a passive RC low pass filter.

**Figure 5:**
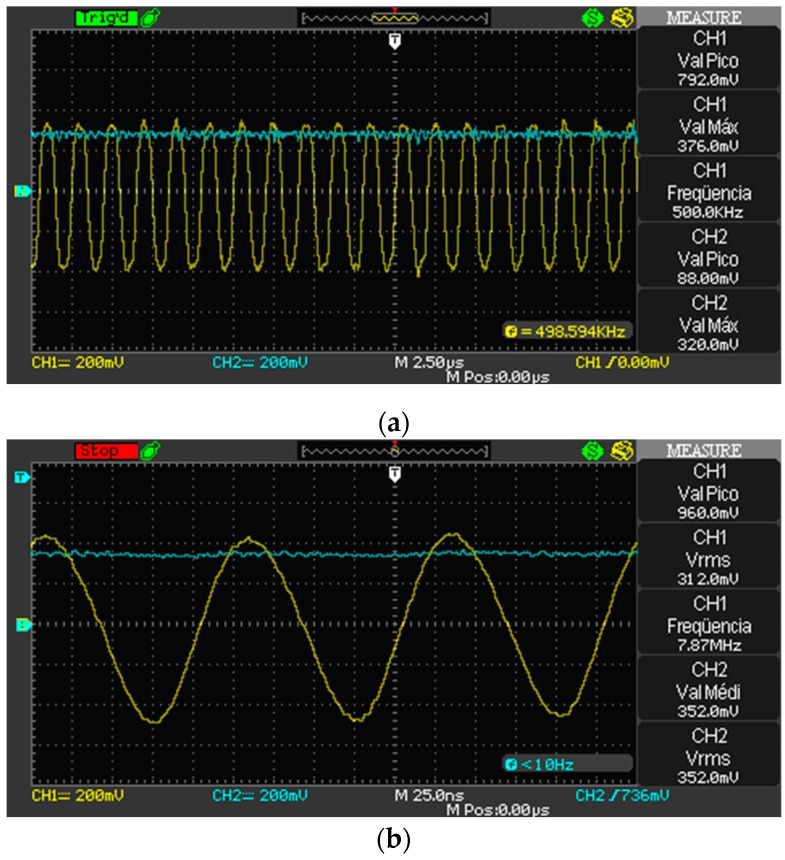
(**a**) 500 kHz and rectified signal; (**b**) 8 MHz and rectified signal.

The filters for both 500 kHz and 8 MHz PWM signals are formed by connecting in series the following steps: (i) two passive 1st order RC low-pass filters, cascaded together to form a 2nd order filter; and (ii) a multiple feedback infinite gain band-pass active filter, like in [31]. For 500 kHz and 8 MHz signals, the cut-off frequencies of each 2nd order low-pass filter are 677 kHz and 9.3 MHz, respectively; the two cut-off frequencies of each band-pass filter are 535.9 kHz and 718.9 kHz, and 5.9 MHz and 14.5 MHz, respectively. Figure 5a,b shows the sinusoidal signals applied to the sensor and the output of the rectifier. The passive RC low pass filter after the rectifier has a cut-off frequency of 10 kHz.

It is possible to use a variety of microcontrollers and amplifiers to implement the proposed circuit. For this implementation, the I-V converter uses an ADA4817-1 FastFET operational amplifier; the microcontroller is a PIC18F25k80; the multiplexers employ a precision CMOS analog switch DG419; and filters and rectifier are implemented with an AD826 operational amplifier.

It is worth pointing out that the microcontroller provides flexibility to the circuit because the frequency of the sensor’s signal source (*V_i_*) can be changed via firmware. In addition, it performs data acquisition, data pre-processing, and data transmission over an XBee network to a computer to figure out the more complex calculation part to achieve σ and ε. Pre-processing step consists in computing the average of ADC results and removing the outliers. The strategy to perform pre- and post-processing using the microcontroller and a computer, respectively, provide energy saving. So, the sensor can be powered by a battery. The measuring circuit consumption is about 130 mA in operation and 1.15 µA in sleep mode. Data transmitting adds an extra current depending on the radio device type (about 66 mA in this work). The microcontroller is responsible for consuming about 29 mA, and the other elements of the circuit such as resistors, capacitors, amplifiers, and CMOS analog switches consume about 101 mA. However, as the system has energy save mode, and the measuring step is performed in about 1 s, the average current consumption is low, as the system can run in sleep mode for a long time (e.g., if the acquisition period is 2 min, the average current consumption is only 1.08 mA).

### 3.2. Calibration

To perform the calibration procedure, we used twelve different substances as references, all of which have both σ and ε known, as shown in Table 1. A Del Lab DL-150P conductivity meter was used to determine the conductivity of the NaCl solutions at 20 °C. The experiments were performed in the laboratory with air conditioning, at an ambient temperature of approximately 20 °C.

After the conductivity measurements of the samples, the sinusoidal signals with frequencies of 500 kHz and 8 MHz were applied by the measurement circuit, to obtain *A*_0_ and *A*_1_, respectively.

**Figure 6:**
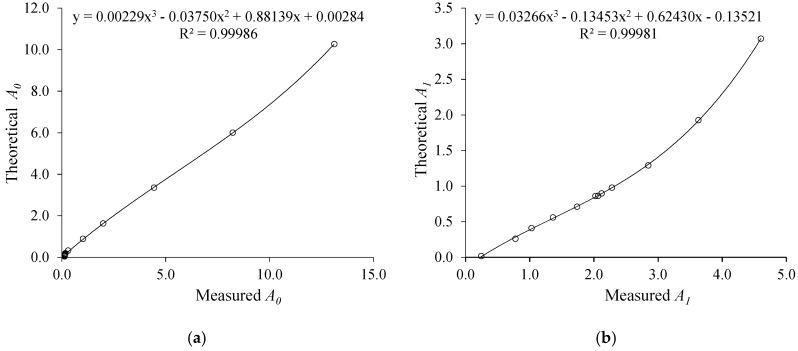
(**a**) Measured and theoretical *A*_0_ relationship; (**b**) Measured and theoretical *A*_1_ relationship.

As a first step to obtain non-biased measurements of σ and ε, we adjust two linear regression models to the gains: measured using the substances of Table 1 and calculated theoretically through (8) and (9), according to Figure 6. Then, we used (10) and (11) to calculate σ and ε of the substances under test. Finally, a linear regression was performed between the measured values and the reference values of the substances.

## 4. Results and Discussion

**Figure 7:**
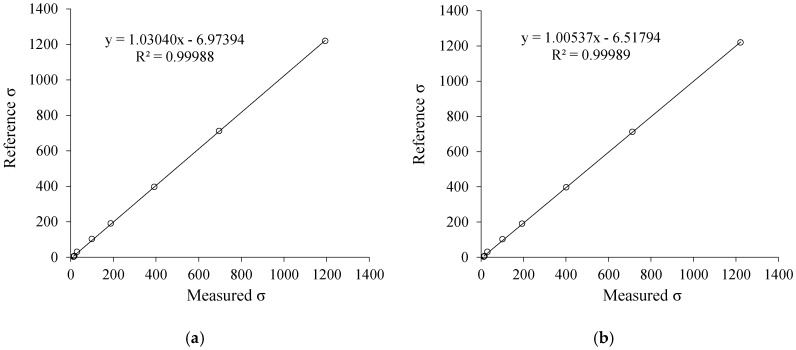
(**a**) Approximation method to estimate σ; (**b**) real-dual-frequency method to estimate σ.

**Figure 8:**
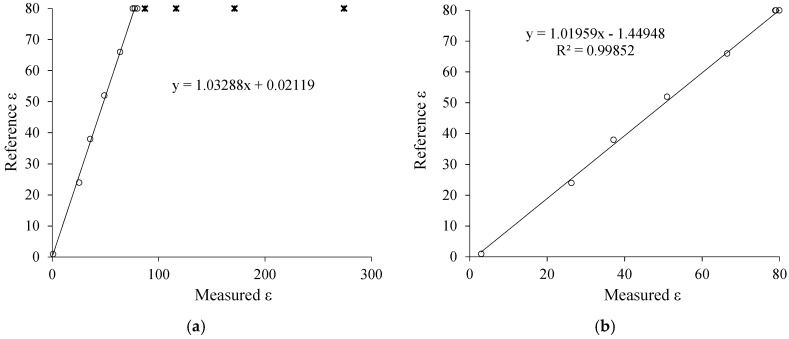
(**a**) Approximation method to estimate ε; (**b**) real-dual-frequency method to estimate ε.

In order to verify the efficiency of the method proposed in this work, Figure 7 and Figure 8 present a comparison between the developed method (real-dual-frequency), which uses (10) and (11), and the approximation method discussed in Reference [2], which uses the approximation of *f* → 0 to estimate σ and *f* → ∞ to estimate ε. Comparing the above mentioned two methods, there is greater accuracy in both σ and ε measurement when using the real-dual-frequency method instead of the frequency approximation method. The comparison can be performed quantitatively by means of the determination coefficient R^2^.

It is noteworthy that the estimation of ε by the approximation method is very compromised when the conductivity is greater than 103.2 μS/cm. Therefore, the model adjusted in Figure 8a does not consider the solutions of NaCl and water ranging from 2 up to 5 (× marks). In Figure 8b it is evident that when using the methodology proposed in this work, it is possible to measure ε in a non-biased way up to 1220 μS/cm, even using lower frequencies when compared to the standard method.

**Figure 9:**
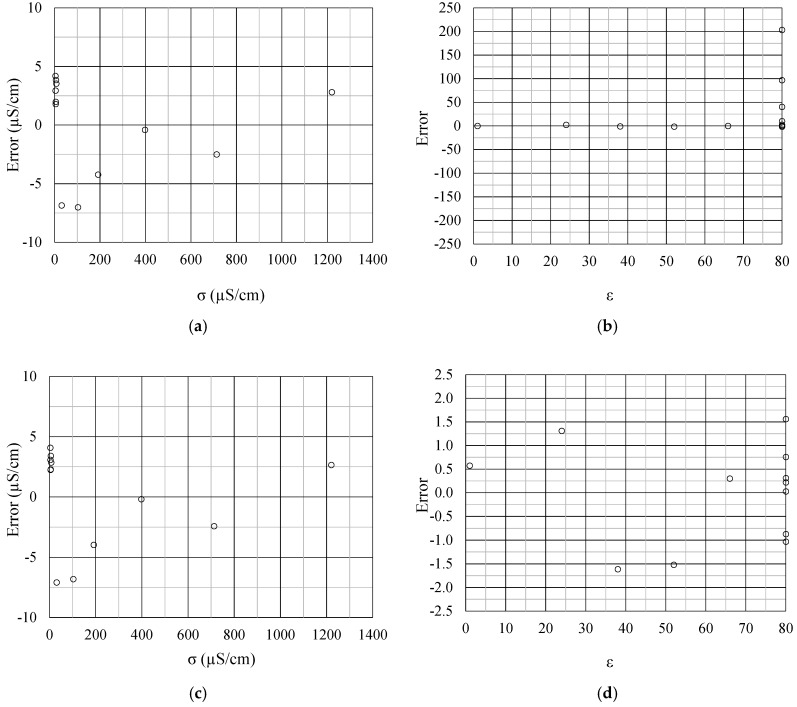
(**a**) Approximation method error curves to estimate σ and (**b**) to estimate ε; (**c**) real dual-frequency method error curves to estimate σ and (**d**) to estimate ε.

Figure 9 shows the measurement errors observed through the two methodologies. Once again, it is evident the advance obtained by using the real-dual-frequency method instead of the approximation of the frequencies (*f* → 0 and *f* → ∞), since there were σ and ε errors reduction, that is, the root mean square errors decreases from 13.75 to 13.47 μS/cm; and from 228.93 to 3.47, respectively. Figure 9b,d shows that the error of ε has been reduced by more than 100 times.

## 5. Conclusions

This paper presents the development of a capacitive impedance measurement system based on an alternative technique to the standard one, which we refer to as real-dual-frequency method. The method consists of measuring two gains of the sensor circuit signal at two different frequencies.

The main advance reported in this work is the use of the frequency response mathematical modeling of the system instead of using the approximation frequencies method to estimate σ and ε. In theory, the approximation method ensures measurement independence between the quantities only if the frequency range of the applied signals is contained within the same frequency range of the two plateaus in the frequency response curve of the system, which may require the application of high frequencies when the medium under test has high conductivity. This method does not eliminate the effect that σ exerts on ε, and vice versa, if the frequencies used are in the decay interval of 20 dB/dec of the frequency response of the system, which in practice happens if the higher frequency is not high enough to reach the second plateau.

By means of the method proposed in this work, real-dual-frequency method, it is possible to perform the measurements of σ and of ε separately, even using frequencies in the decay interval of 20 dB/dec, i.e., frequencies lower than the second frequency of the system. The separation of the effects was obtained in the tests carried out in this work for conductivity up to 1220 μS/cm.

The proposed system showed a maximum error of 0.6% for the measurement of electrical conductivity and 2% for the relative dielectric constant, considering measurement ranges from 0 to 1220 μS/cm and from 1 to 80, respectively.

We highlight that this method can be applied for measuring materials impedance with higher conductivity than 1220 μS/cm by increasing the operational amplifiers voltage rail supplies and/or by decreasing the voltage applied to the sensor and increasing the ADC resolution.

## Figures and Tables

**Table 1:** Substances used in the calibration process.

Substance	σ (µS/cm)	ε
Air	0	1
Solution of water and NaCl 1	103.2	80
Solution of water and NaCl 2	191.8	80
Solution of water and NaCl 3	397.6	80
Solution of water and NaCl 4	712.9	80
Solution of water and NaCl 5	1220	80
Distillated water	7.850	80
75% of water and 25% of ethanol	5.730	66
50% of water and 50% of ethanol	3.979	52
25% of water and 75% of ethanol	4.327	38
Ethanol	5.179	24
Drinking water	31.19	80
