# Erratum: Rêgo Segundo, A.K.; et al. Capacitive Impedance Measurement: Dual-Frequency Approach. *Sensors* 2019, *19*, 2539

**DOI:** 10.3390/s21062149

**Published:** 2021-03-19

**Authors:** Alan Kardek Rêgo Segundo, Érica Silva Pinto, Gabriel Almeida Santos, Paulo Marcos de Barros Monteiro

**Affiliations:** 1 Escola de Minas, Universidade Federal de Ouro Preto (UFOP), Morro do Cruzeiro, 35400-000 Ouro Preto, MG, Brazil; erica.lins15@gmail.com (É.S.P.); gabrielalmeida.el@gmail.com (G.A.S.); pmemop@gmail.com (P.M.d.B.M.); 2 Instituto Tecnológico Vale (ITV), Avenida Juscelino Kubitschek, 31, Bauxita, 35400-000 Ouro Preto, MG, Brazil

## Text Correction

There was two errors in the original article [1].

1. On page 3, instead of “The real parts of (1) and (2) are related to the losses by Joule effect.”, it should read: “The real part of (3) is related to the losses by Joule effect“.

A correction has been made to *Section 2. Theory, 3rd Paragraph*:

The impedance (**Z**) of a material corresponds to the ratio between the voltage (**V**) and the current (**I**) phasors, according to Ohm’s law in complex notation, that is

(3) Z=R+jX

where *R* is the resistance (Ω) and *X* the reactance (Ω). The real part of (3) is related to the losses by Joule effect. The imaginary part is the ability to exchange energy.

2. On page 5, both Equations (8) and (9) should have $C_{f}^{2}$ instead of $C_{x}^{2}$ in the denominator.

A correction has been made to *Section 2. Theory, Equations (8) and (9)*:

(8) $$A_{0}=\sqrt{\frac{G_{x}^{2}+\omega_{0}^{2}C_{x}^{2}}{G_{f}^{2}+\omega_{0}^{2}C_{f}^{2}}}$$

(9) $$A_{1}=\sqrt{\frac{G_{x}^{2}+\omega_{1}^{2}C_{x}^{2}}{G_{f}^{2}+\omega_{1}^{2}C_{f}^{2}}}$$

The authors apologize for any inconvenience caused and state that the scientific conclusions are unaffected. The original article has been updated.

## References

[1] Rêgo Segundo A.K., Silva Pinto É., Almeida Santos G., de Barros Monteiro P.M. (2019). Capacitive Impedance Measurement: Dual-frequency Approach. Sensors.

